# Supplementary material for: Hsp70 in Liquid Biopsies—A Tumor-Specific Biomarker for Detection and Response Monitoring in Cancer
Source: Cancers (Basel). 2021 Jul 23;13(15):3706. doi: 10.3390/cancers13153706 (PMC8345117; doi:10.3390/cancers13153706)
Supplement: Supplementary file 1 [file cancers-13-03706-s001.zip › cancers-1248171-supplementary.pdf]

Supplementary Materials

Hsp70 in Liquid Biopsies—A Tumor-Specific Biomarker for Detection and Response Monitoring in Cancer

Caroline Werner, Stefan Stangl, Lukas Salvermoser, Melissa Schwab, Maxim Shevtsov, Alexia Xanthopoulos, Fei Wang, Ali Bashiri Dezfouli, Dennis Thölke, Christian Ostheimer, Daniel Medenwald, Martin Windberg, Matthias Bache, Martin Schlapschy, Arne Skerra and Gabriele Multhoff

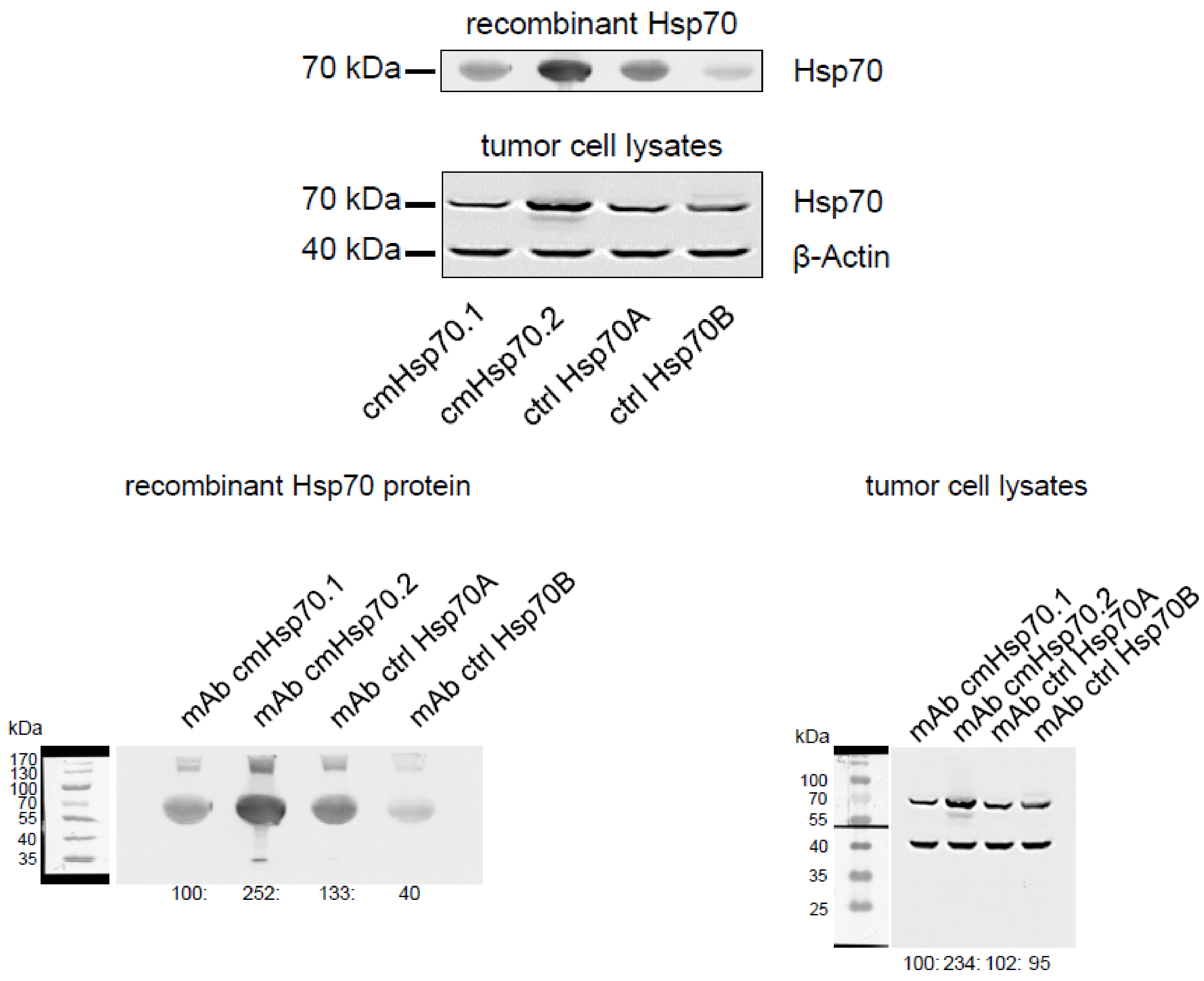

Detailed information about Figure 2a.

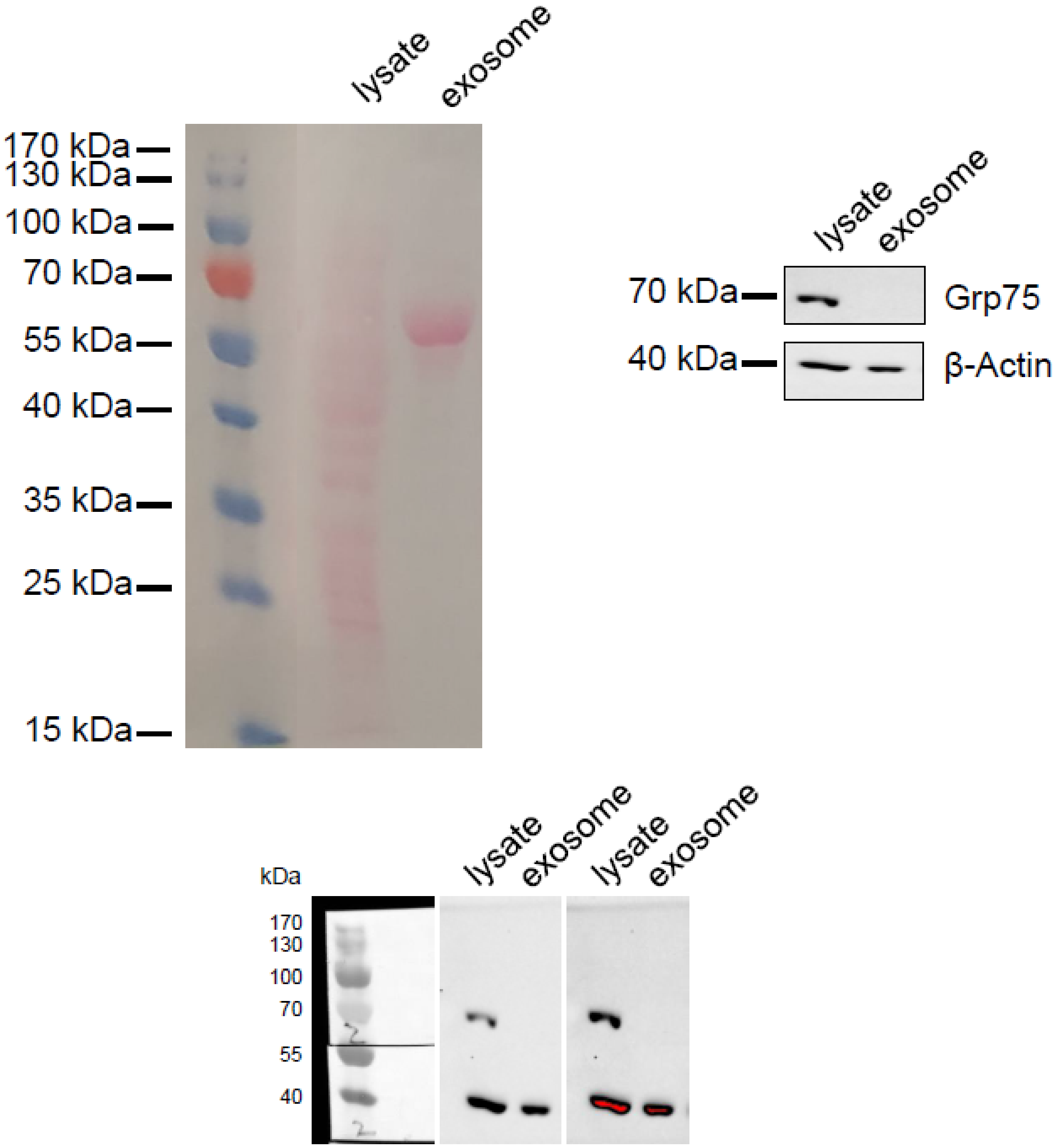

Detailed information about Figure 6f.

© 2021 by the authors. Licensee MDPI, Basel, Switzerland. This article is an open access article distributed under the terms and conditions of the Creative Commons Attribution (CC BY) license (http://creativecommons.org/licenses/by/4.0/).
